# Atypical Renal Hypoplasia in HNF1B-Related Disease: The Clinical Impact of Early Genetic Diagnosis

**DOI:** 10.3390/jcm15197537

**Published:** 2026-09-28

**Authors:** Filip Koszałka, Aleksandra Gałan, Amir Nour Mohammadi, Jagoda Góra, Izabela Zakrocka, Agnieszka Korolczuk, Wojciech Załuska

**Affiliations:** 1 Students’ Scientific Club of Nephrology and Clinical Transplantology, Medical University of Lublin, Jaczewskiego 8, 20-090 Lublin, Poland; filipkoszalka6@outlook.com (F.K.); aleksandra.galan00@gmail.com (A.G.); amir.nm2001@gmail.com (A.N.M.); jagoda1436@gmail.com (J.G.); 2 Department of Nephrology, Medical University of Lublin, Jaczewskiego 8, 20-090 Lublin, Poland; wojciech.zaluska@umlub.edu.pl; 3 Department of Clinical Pathomorphology, Medical University of Lublin, Jaczewskiego 8b, 20-090 Lublin, Poland; agnieszka.korolczuk@umlub.edu.pl

**Keywords:** *HNF1B*, ADTKD-HNF1B, RCAD, MODY5, renal hypoplasia, chronic kidney disease, genotype–phenotype correlation, next-generation sequencing

## Abstract

**Background:** Genetic testing is an increasingly important component in the diagnostic evaluation of kidney diseases. Pathogenic variants in the *HNF1B* gene cause a disorder classically known as renal cysts and diabetes (RCAD) syndrome, typically characterised by bilateral renal cysts, diabetes mellitus, and a broad spectrum of other extrarenal abnormalities. However, non-cystic presentations often lead to diagnostic delays. **Case Report:** We report the case of a 28-year-old woman evaluated for chronic kidney disease of unknown aetiology who presented with atypical radiological findings. Imaging revealed bilateral renal hypoplasia completely devoid of cystic transformation. The patient exhibited no proteinuria, electrolyte or acid–base disturbances, diabetes mellitus, or pancreatic abnormalities. Next-generation sequencing identified pathogenic *HNF1B* missense variant (p.Met160Val), confirming the diagnosis of genetic kidney disease. The patient’s clinical course remained stable throughout the follow-up period. **Conclusions:** This case illustrates the marked phenotypic heterogeneity of HNF1B-associated disease and supports incorporating genetic testing in patients with unexplained structural kidney abnormalities, even in the absence of classical cystic renal disease or typical extrarenal manifestations, to ensure accurate diagnosis and treatment, and to facilitate appropriate family counselling.

## 1. Introduction

Genetic causes of kidney disease are increasingly recognised, with 10–30% of adults having chronic kidney disease (CKD) secondary to a single gene mutation [1]. Owing to the wider availability of next-generation sequencing and greater awareness among healthcare professionals, genetic work-ups for patients with kidney disorders have become more a routine practice [2]. The primary indications for genetic evaluation include a positive family history, extrarenal features, atypical clinical or radiological findings, and resistance to standard treatment. Early diagnosis of a genetic kidney disease facilitates the introduction of targeted treatments, reduces unnecessary medical interventions, and allows for appropriate prognostic and family counselling.

HNF1B-associated disease—historically known as renal cysts and diabetes (RCAD) syndrome and classified by some authors within the autosomal dominant tubulointerstitial kidney disease (ADTKD) spectrum—is caused primarily by pathogenic variants of the hepatocyte nuclear factor 1 beta (*HNF1B*) gene located on chromosome 17q12 [3]. *HNF1B* gene encodes a transcription factor that is essential for the embryonic development of the kidneys, pancreas, liver, and urogenital tract [4]. Mutations in this gene represent one of the most common monogenic causes of developmental kidney abnormalities and are increasingly recognised as major contributors to congenital anomalies of the kidney and urinary tract (CAKUT).

The clinical features of HNF1B-associated disease are highly diverse, though renal manifestations remain the most common [5]. The classical renal phenotype is characterised by bilateral kidney cysts, cystic dysplasia, or hyperechogenic kidneys [6]. However, affected individuals may also present with non-cystic structural abnormalities, such as renal hypoplasia, a single-functioning kidney, or near-normal imaging accompanied by impaired renal function [7]. Extrarenal features are equally varied and can include maturity-onset diabetes of the young type 5 (MODY5), pancreatic hypoplasia, early-onset gout secondary to hyperuricaemia, hypomagnesaemia, and urogenital tract malformations [8]. The severity of renal impairment ranges widely, from mild CKD to end-stage renal disease requiring kidney replacement therapy.

Diagnosing HNF1B-associated disease can be highly challenging due to this vast phenotypic spectrum and the potential for isolated kidney involvement without any systemic symptoms. In the absence of obvious cystic kidney malformations or diabetes mellitus, recognition of this genetic condition is frequently delayed, and the patient may be misclassified as having hypertensive CKD or CKD of unknown aetiology (CKDx). Consequently, genetic testing for *HNF1B* variants is paramount not only for establishing the diagnosis but also for guiding clinical management and prognosis. Clinicians should be able to recognise atypical, non-cystic presentations, such as isolated renal hypoplasia, to prevent underdiagnosis and afford timely, personalised intervention.

The primary aim of this report is to present the case of a young woman with CKDx who exhibited an isolated kidney damage phenotype. By detailing her atypical presentation—bilateral renal hypoplasia completely devoid of cystic changes or extrarenal manifestations—and her subsequent diagnosis via next-generation sequencing, we aim to highlight the marked phenotypic heterogeneity of HNF1B-associated disease. This case underscores the conclusion that genetic testing should be integrated into the evaluation of unexplained structural kidney abnormalities, even when classical cystic or systemic features are entirely absent.

## 2. Clinical Case Description

A 28-year-old woman with a two-year history of CKD of unknown aetiology (CKDx) was referred to our nephrology clinic for further evaluation. She reported experiencing sporadic pain in the small joints of her hands but denied any systemic complaints or joint oedema suggestive of an active inflammatory process. Her past medical history was unremarkable, with no previous hospitalisations. Her family history was notable for kidney failure in her maternal grandmother; however, no further clinical or genetic details regarding this relative were available. At the time of this report, none of the patient’s first-degree relatives was diagnosed with kidney disorder and none had undergone genetic testing for the identified variant, leaving its inherited versus *de novo* origin undetermined.

On physical examination, the patient was in good general condition and haemodynamically stable, with normal cardiovascular and respiratory findings. No peripheral oedema or cutaneous abnormalities were observed. Her urine output was normal, and she reported no dysuric symptoms. Routine urinalysis was entirely unremarkable, showing no proteinuria, and microscopic examination of the urine sediment was inactive, with no evidence of erythrocyturia or cellular casts.

Laboratory investigations revealed mildly impaired renal function, with an elevated serum creatinine of 1.2 mg/dL (reference range: 0.51–0.95 mg/dL), urea of 51.6 mg/dL (reference range: 17–43 mg/dL), and blood urea nitrogen of 24.1 mg/dL (reference range: 7–18.8 mg/dL). Serum electrolytes, parathyroid hormone levels, and blood gas analysis parameters were within reference limits. A complete blood count was largely unremarkable, save for a marginal decrease in mean corpuscular haemoglobin (26.6 pg; reference: >27 pg) and a mild increase in monocyte percentage, although the absolute monocyte count was normal. Serum magnesium and uric acid levels were within reference limits. The levels of immunoglobulins (IgA, IgG, and IgM) and complement factors (C3 and C4) were within normal ranges.

Autoimmune screening yielded a positive antinuclear antibody (ANA) titre between 1:100 and 1:160 with a speckled pattern. Subsequent immunoblotting identified specific antibodies against nuclear ribonucleoprotein (nRNP/Sm), Mi-α, and centromere protein A (CENP A), alongside borderline positive results for Ku, Smith (Sm) proteins, and a subunit of RNA polymerase III (RP11). However, due to the absence of an active urinary sediment (e.g., proteinuria, erythrocyturia) and the lack of systemic clinical features of connective tissue disease, these serological findings were considered non-specific and unrelated to the structural renal abnormalities. Additional screens for anti-liver-kidney microsome (anti-LKM), antimitochondrial (AMA), and anti-smooth muscle (ASMA) antibodies were negative. A dry blood spot test for Fabry disease was also negative, showing normal alpha-galactosidase activity (6.5 micromol/L/h; reference: >2.8 micromol/L/h) and lyso-GL-3 concentration (1.6 ng/mL; reference: <3.5 ng/mL).

Abdominal ultrasonography performed prior to hospitalisation demonstrated bilateral small kidneys without cystic transformation or other structural abnormalities (Figure 1). The right kidney measured 87.5 × 33.5 mm, and the left measured 85 × 31.5 mm. Both kidneys were in a normal anatomical position with preserved corticomedullary differentiation and no evidence of hydronephrosis, nephrolithiasis, or increased echogenicity. Other abdominal organs, including the liver, gallbladder, pancreas, spleen, and urinary bladder, appeared normal, and the abdominal aorta was not dilated.

Subsequent abdominal magnetic resonance imaging (MRI) confirmed the reduced kidney size but revealed no other significant pathologies within the urinary tract or pancreas (Figure 2). To further differentiate between congenital renal hypoplasia and acquired renal atrophy, dynamic renal scintigraphy utilising technetium-99m ethylenedicysteine (99mTc-EC) was performed. The study revealed smooth kidney contours with uniform radiotracer distribution throughout the parenchyma bilaterally. Relative renal function was preserved and symmetrical, calculated at 50.7% for the left kidney and 49.3% for the right. Both kidneys demonstrated normal secretory and excretory function, with a normal time-to-peak activity (Tmax = 3.0 min) bilaterally. Due to the small size of the kidneys and the absence of nephrotic or nephritic syndrome features, a kidney biopsy was not performed.

Given the persistent renal impairment of unclear origin, next-generation sequencing was performed using the NovaSeq X platform (Illumina, San Diego, CA, USA). The genetic analysis evaluated the coding exons and their flanking intronic sequences. The mean sequencing depth was 217.7, with 98.9% of the target sequences meeting the established quality threshold. Copy-number variant (CNV) analysis was not performed as part of this targeted panel. This genetic analysis revealed a pathogenic heterozygous missense variant in the *HNF1B* gene (p.Met160Val, NM_000458.4: c.478A>G). This variant was classified as pathogenic in accordance with ACMG/AMP guidelines, supported by its absence in large population databases (PM2), multiple lines of computational evidence indicating a deleterious effect on the gene product (PP3), and its previous identification in multiple independent affected individuals (PS4). No other pathogenic variants were detected. Following the diagnosis, the patient remained clinically stable and exhibited no extrarenal manifestations commonly associated with HNF1B-associated disease, such as diabetes mellitus, pancreatic anomalies, or electrolyte disturbances. She was discharged with recommendations for regular nephrological follow-up and genetic counselling for her family members.

## 3. Discussion

CKDx remains a diagnostic challenge, particularly when standard clinical, biochemical, and imaging evaluations fail to identify an underlying aetiology. Increasing evidence demonstrates that genetic factors, especially pathogenic variants in genes such as *HNF1B*, can underlie subtle or atypical structural kidney abnormalities, including renal hypoplasia or even normal-appearing kidneys with functional impairment. Recognising these non-classical presentations matters, as they frequently lead to delayed diagnoses or misclassification as idiopathic CKD. Incorporating genetic testing into the evaluation of CKD can therefore clarify the underlying aetiology and guide clinical management.

Contrary to the predominant phenotypes described in large cohorts and case series (Table 1), our patient exhibited bilateral renal hypoplasia without cystic transformation or extrarenal involvement. However, atypical non-cystic presentations are increasingly recognised as important subsets of this disorder. For instance, Lyu et al. [9] recently described a family harbouring a 17q12 microdeletion presenting with unilateral renal agenesis and mild hydronephrosis completely devoid of cysts. Similarly, Gambella et al. [10] emphasised that the disease spectrum is broader than previously assumed, and Valpaços et al. [11] highlighted the diagnostic challenges when cystic morphology is not readily evident on ultrasound, which supports the case for genetic evaluation in such scenarios.

The absence of extrarenal features in our patient further illustrates the heterogeneity of HNF1B-related disorders. Although diabetes mellitus is a common feature of HNF1B-associated disease, affecting up to 50% of patients, its onset may occur later in life, and some carriers remain normoglycaemic. Pancreatic or genital tract anomalies, as well as electrolyte disturbances such as hypomagnesaemia, may also develop progressively, highlighting the importance of long-term surveillance.

Genetic testing remains a vital diagnostic tool for patients with kidney disorders, especially in cases such as ours where imaging findings are atypical and inconclusive. Next-generation sequencing confirmed the presence of a pathogenic *HNF1B* gene variant, which aligns with recommendations from recent reviews advocating early genetic screening for patients with bilateral renal anomalies or CKDx. Timely identification enables proactive management, including the regular monitoring of renal function, screening for metabolic disturbances, and initiating genetic counselling for family members. Furthermore, emerging research on biomarkers and metabolic pathways, such as the elevated lysophosphatidic acid levels described by Małachowska et al. [15], may provide additional tools to refine phenotypic risk assessment for patients with *HNF1B* gene mutations in the near future.

HNF1B-associated disease is also characterised by a distinctive biochemical signature that can support the diagnosis even when renal imaging is ambiguous. Hyperuricaemia, often disproportionate to the degree of renal impairment, has been described in a substantial proportion of patients and is recognised as an early, relatively common, albeit non-specific feature. Hypomagnesaemia due to renal magnesium wasting occurs with similar frequency [16]. Systematically documenting these parameters alongside standard electrolyte and parathyroid hormone assessments may help distinguish HNF1B-associated disease from other causes of unexplained structural kidney disease at initial presentation. Mechanistically, *HNF1B* functions as a POU-homeodomain transcription factor. Its DNA-binding region comprises a bipartite structure: an N-terminal POU-specific subdomain and a C-terminal homeodomain connected by a flexible linker, which together mediate sequence-specific DNA binding and homodimerisation or heterodimerisation with HNF1A protein [4,17]. Residue 160 lies within this POU-specific subdomain. In vitro studies of a different substitution at this exact position (p.Met160Thr) revealed that the mutant protein does not act via a complete dominant-negative mechanism; instead, it maintains some DNA-binding ability, suggesting haploinsufficiency as the primary driver of the phenotype [18]. This structural context may help explain why substitutions at this position, including the p.Met160Val variant identified in our patient, are compatible with a comparatively mild and variable renal phenotype, in contrast to the more severe, early-onset presentations associated with shortening or DNA-contact-disrupting *HNF1B* variants.

Notably, the specific variant identified in our patient, p.Met160Val, is not a novel finding. The exact substitution was previously reported in five patients from two independent families: three affected individuals in a family investigated for early and severe polycystic kidney disease [18,19], and a mother and son diagnosed with HNF1B-associated disease via whole-exome sequencing [20]. Four of these five previously reported patients had small renal cysts, and the two who developed diabetes did so later in adolescence (both at 17 years of age), with no pancreatic morphological abnormalities described in any case. Therefore, our patient’s presentation of bilateral renal hypoplasia entirely without cystic changes and no diabetes at 28 years of age represents the first reported purely non-cystic phenotype associated with the p.Met160Val variant. This clinical contrast among individuals harbouring the exact same substitution reinforces that there is no clear genotype–phenotype correlation in *HNF1B*-associated disease, which is consistent with haploinsufficiency as the primary disease mechanism. Furthermore, while the condition exhibits autosomal dominant inheritance, mutations and whole-gene deletions frequently occur spontaneously; thus, classical family history of renal disease or diabetes mellitus may be entirely absent. Our patient’s presentation—bilateral renal hypoplasia entirely without cystic changes and no diabetes at 28 years of age—extends rather than establishes the phenotypic spectrum associated with this variant. It reinforces the concept that even a previously characterised *HNF1B* variant can produce a substantially divergent, non-cystic renal phenotype. A recent case report describing a different substitution at the same codon, p.Met160Thr, similarly demonstrated marked functional and clinical differences from p.Met160Val despite affecting the identical residue, which points to the value of residue-level rather than gene-level genotype–phenotype correlations in HNF1B-associated disease [18].

## 4. Conclusions

This case supports the role of genetic testing in identifying HNF1B-associated disease and highlights the need for greater clinical awareness of its atypical forms. Recognising such variability helps ensure accurate diagnosis of renal manifestations, prevent unnecessary interventions, and guide personalised follow-up for patients and their families. HNF1B-associated disease should remain part of the differential diagnosis for unexplained structural kidney disease, even in the absence of cystic changes, diabetes, or other extrarenal features. Genetic testing can prove definitive in such atypical, non-classical presentations.

## Figures and Tables

**Figure 1:**
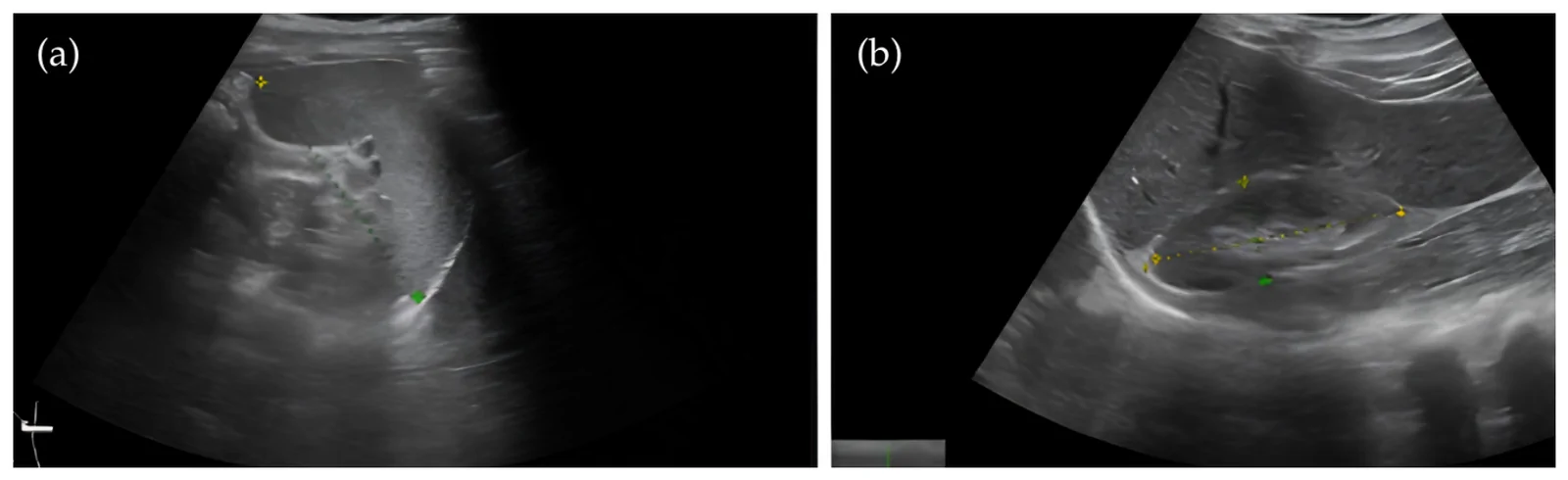
Abdominal ultrasonography (USG): (**a**) left kidney, measuring 85 • 31.5 mm, (**b**) right kidney, measuring 87.5 • 33.5 mm. Yellow and green markers indicate caliper measurement points used to determine renal length.

**Figure 2:**
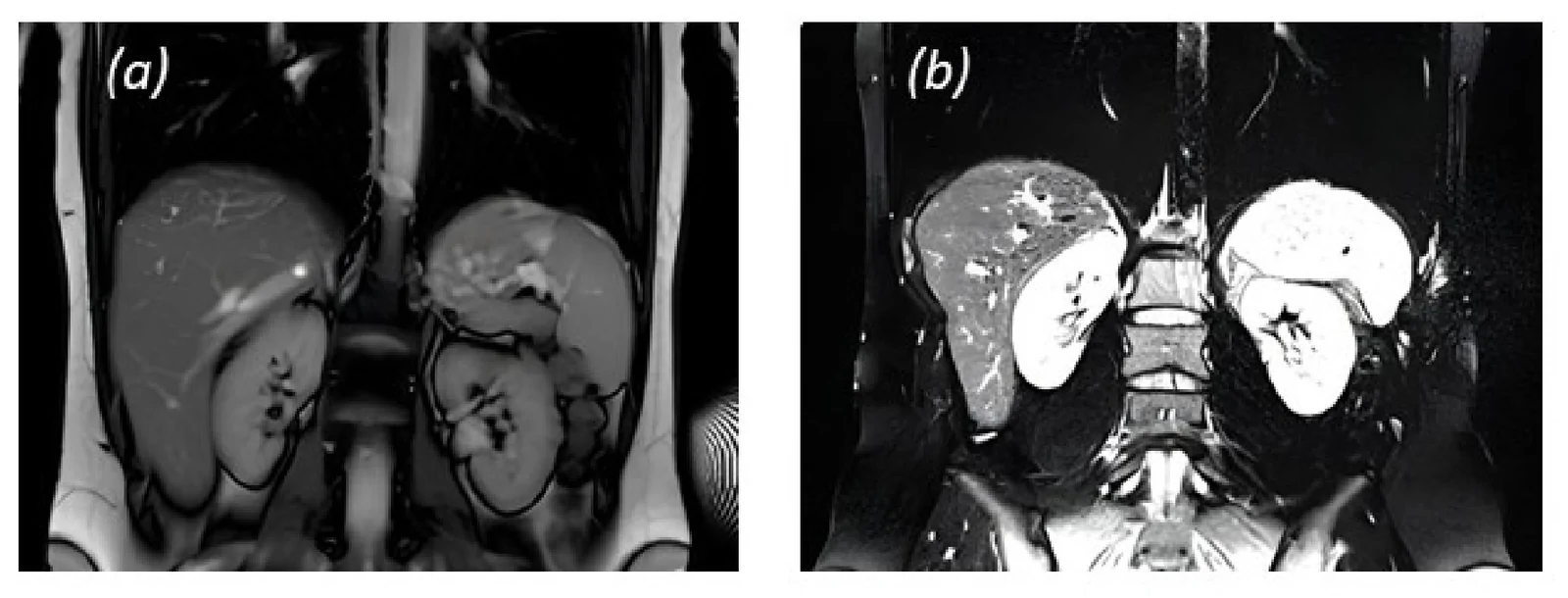
Magnetic resonance imaging (MRI) of the abdomen, coronal plane. (**a**) T1- and (**b**) T2- weighted imaging.

**Table 1:** Kidney manifestations of *HNF1B* gene mutation in large cohorts and case series.

References	Population	Kidney Phenotype
Bingham C. et al. [8]	55 patients with *HNF1B* mutations	66% of patients had kidney cysts
Buffin-Meyer B et al. [12]	521 patients with *HNF1B* variants	60% had renal cortical cysts
		20% with hyperechogenic kidneys
		7% had multicystic and dysplastic kidney
		5.5% with point mutation had kidney hypoplasia
Lim SH et al. [13]	14 paediatric patients with *HNF1B* mutations	86% had multiple kidney cysts
		64% had renal parenchymal hyperechogenicity
		29% had unilateral multicystic and dysplastic kidney
		21% had hydronephrosis
		14% had vesicoureteral reflux
Izzi C et al. [14]	13 affected patients (from 7 probands) with pathogenic variants	46% had normal size kidneys
		31% had several bilateral cysts
		8% had multiple bilateral cysts
		8% few unilateral cysts
		8% had enlarged kidneys
		8% had hypoplasia of the right kidney

## Data Availability

The data supporting the findings of this case report are available from the corresponding author upon reasonable request. The data are not publicly available due to privacy restrictions, as they contain information that could compromise the identity of the patient.

## Abbreviations

The following abbreviations are used in this manuscript:

ADTKD	Autosomal dominant tubulointerstitial kidney disease
AMA	Antimitochondrial antibodies
ANA	Antinuclear antibodies
Anti-LKM	Anti-liver-kidney microsome antibodies
ASMA	Anti-smooth muscle antibodies
C3	Complement component 3
C4	Complement component 4
CAKUT	Congenital anomalies of the kidney and urinary tract
CENP A	Centromere protein A
CKD	Chronic kidney disease
CKDx	Chronic kidney disease of unknown aetiology
DNA	Deoxyribonucleic acid
HNF1B	Hepatocyte nuclear factor 1 beta
IgA	Immunoglobulin A
IgG	Immunoglobulin G
IgM	Immunoglobulin M
lyso-GL-3	Globotriaosylsphingosine
MCH	Mean corpuscular haemoglobin
MODY5	Maturity-onset diabetes of the young type 5
MRI	Magnetic resonance imaging
nRNP/Sm	Nuclear ribonucleoprotein
RCAD	Renal cyst and diabetes
RP11	RNA polymerase III subunit
Sm	Smith proteins
USG	Ultrasonography
